# Phenotypic stratification of Low-grade Glioma using multimodal MRI via outcome-weighted integrative clustering

**DOI:** 10.1186/s12883-025-04420-0

**Published:** 2025-11-04

**Authors:** Qi Yang, Gaiqin Liu, Tong Wang, Zhaoyang Xu, Junyu Yan, Ruiling Fang, Yanhong Luo, Hongmei Yu, Yan Tan, Hui Zhang, Guoqiang Yang, Hongyan Cao

**Affiliations:** 1 Department of Health Statistics, Shanxi Provincial Key Laboratory of Major Diseases Risk Assessment, School of Public Health, Shanxi Medical University, Taiyuan, 030001 China; 2 MOE Key Laboratory of Coal Environmental Pathogenicity and Prevention, Shanxi Medical University, Taiyuan, 030001 China; 3 Academy of Medical Sciences, Shanxi Medical University, Taiyuan, 030001 China; 4 Department of Radiology, First Hospital of Shanxi Medical University, Taiyuan, 030001 China; 5 College of Medical Imaging, Shanxi Medical University, Taiyuan, 030001 China

**Keywords:** Phenotypic subtype, Low-grade glioma, Magnetic resonance image, Outcome weighted integrative clustering, SurvClust

## Abstract

**Background:**

Low-grade glioma (LGG) is a diverse group of primary brain tumors, whose molecular heterogeneity hinders classification by traditional pathological methods. Accurate phenotypic subtyping of LGG is essential for capturing tumor characteristics and optimizing clinical management. We intend to identify LGG phenotypic subtypes based on multimodal magnetic resonance imaging (MRI) data, enhancing prognosis evaluation and optimizing treatment strategy.

**Methods:**

This was a retrospective multicenter study, and data were drawn from the First Hospital of Shanxi Medical University (FHSXMU) and Shanxi Provincial People’s Hospital (SPPH) (FHSXMU/SPPH cohort, *n* = 162), and The Cancer Genome Atlas (TCGA)/The Cancer Imaging Archive (TCIA) (TCGA/TCIA cohort, *n* = 118). In the FHSXMU/SPPH cohort, LGG phenotypic subtypes were identified using the outcome-weighted integrative clustering method (survClust) based on multimodal MRI data (CE-T1 and T2FLAIR). A multivariate Cox proportional hazards model was applied to evaluate survival differences between subtypes. Statistical comparisons between subtypes were performed, and the statistically significant MRI features were utilized to predict clinically relevant biomarkers – isocitrate dehydrogenase (IDH) mutation combined with O6-methylguanine-DNA methyltransferase (MGMT) promoter methylation. Five models were constructed, including fused kernel partial least squares with the genetic algorithm (GA-fKPLS), logistic regression, random forest, support vector machine, and *k*-nearest neighbor. In the TCGA/TCIA cohort, we validated the identified phenotypic subtypes and further explored their biological characteristics by analyzing pathway activity and immune infiltration levels using mRNA expression data.

**Results:**

Two distinct LGG phenotypic subtypes were identified in the FHSXMU/SPPH cohort, and validated in the TCGA/TCIA cohort. In the FHSXMU/SPPH cohort, significant differences in pathological grade, MGMT promoter status, IDH genotype, survival status, tumor volume, and survival outcome (*HR*: 2.553, 95%*CI*: [1.226–5.315]) between the two subtypes (*P* < 0.05). Compared to other four models, the GA-fKPLS model exhibited superior predictive performance (AUC: 0.809). In the TCGA/TCIA cohort, two LGG phenotypic subtypes showed significant differences in pathway activities (JAK-STAT, TNF-α, p53) and immune cell infiltration (M2 macrophages, T cell regulatory, Monocytes) (*P*_adj_ < 0.05).

**Conclusion:**

This study identified two LGG phenotypic subtypes and potential biomarkers, offering supplementary information for clinical evaluation and treatment decision-making.

**Graphical Abstract:**

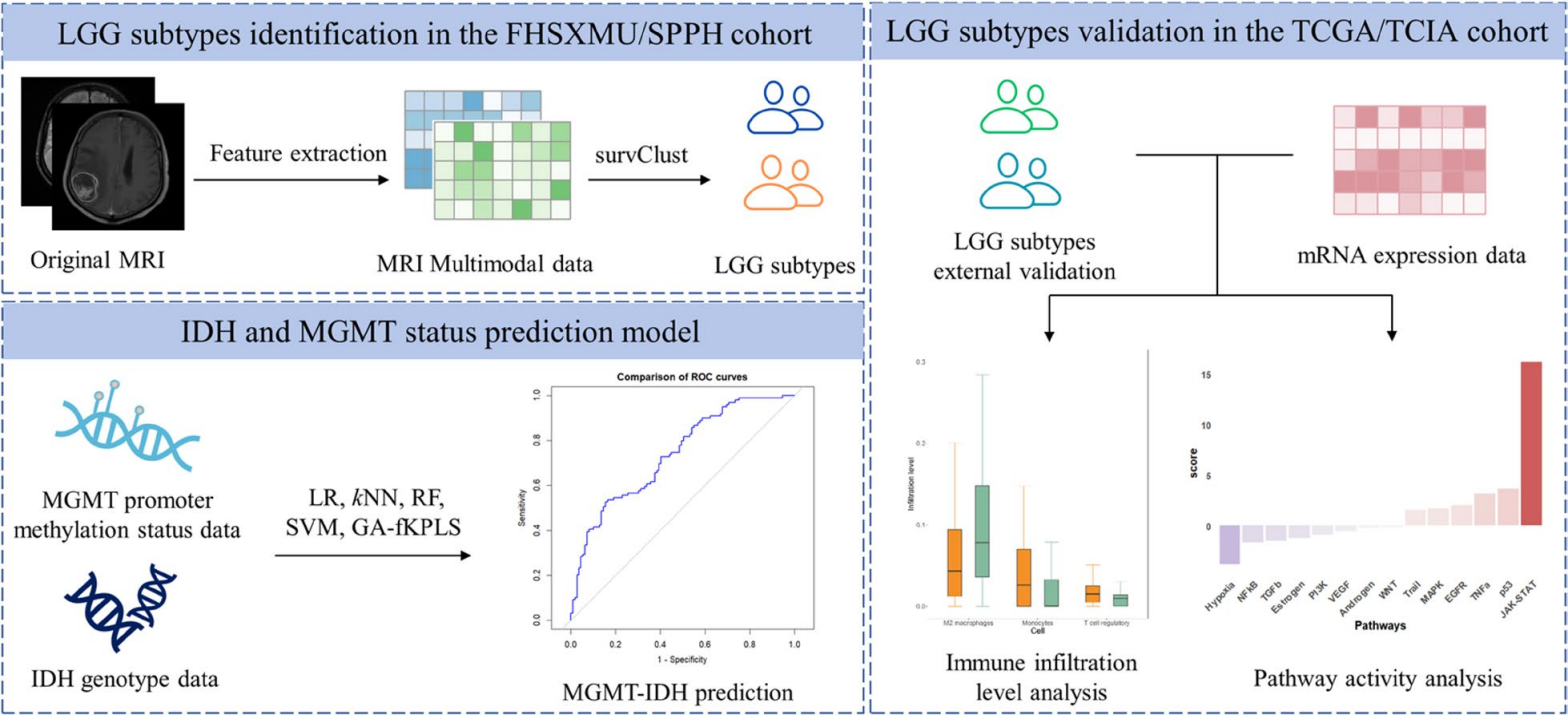

**Supplementary Information:**

The online version contains supplementary material available at 10.1186/s12883-025-04420-0.

## Background

Low-grade gliomas (LGG), defined as World Health Organization (WHO) grades II and III gliomas, are the most common diffuse and infiltrative primary malignant brain tumors in adults [1–3]. The histopathological classification of LGG has traditionally guided clinical management decisions. However, due to the high heterogeneity of tumors, even when LGG patients at the same histological stages receive the same treatment, their responses and clinical outcomes can vary widely [4, 5]. There is an urgent need to employ robust methods that can identify high-risk LGG phenotypic subtypes and uncover potential therapeutic targets, facilitating precision diagnosis, intervention, and clinical decision-making for LGG patients.

Magnetic resonance imaging (MRI) is commonly used for preoperative evaluation of LGG due to its non-invasive nature and widespread clinical application [6]. MRI encompasses modalities such as post-contrast enhanced T1-weighted (CE-T1) imaging and T2-weighted fluid attenuation inversion recovery (T2FLAIR) imaging, each of which can provide distinct information about the tumor. CE-T1 enables precise tumor localization and size assessment by enhancing the visibility of angiogenesis-associated abnormal areas using contrast agents like gadolinium. CE-T1 is particularly useful in identifying and delineating the boundaries of LGG lesions, especially for surgical planning and tumor volumetry [7]. T2FLAIR combines T2-weighted imaging with fluid-attenuated inversion recovery sequences, effectively displaying edema and abnormal signals in surrounding tissues [8]. It is particularly suited for visualizing the infiltrative growth patterns around tumors and their boundary with normal brain tissue, facilitating the evaluation of tumor extent and potential surgical margins. While each MRI modality has specific advantages in LGG characterization, the multimodal MRI data integration enables more comprehensive tumor profiling, facilitating development of precise individualized treatment strategies and providing reliable prognostic evaluation [9, 10].

Current glioma subtype identification primarily employs conventional clustering methods (e.g., k-means, hierarchical clustering) [11–13]. Multimodal data integration clustering methods allow joint analysis of multimodal data, enhancing information utilization for more comprehensive biological insights [14]. Among existing multimodal data integration methods, most fail to model patient survival outcomes as the optimization objective, instead evaluating prognostic differences through post hoc subtype analysis [15, 16]. This results in the identified molecular subtypes potentially lacking survival differences, thereby limiting their clinical translational value. Unlike traditional multimodal data integration clustering methods, survClust is an outcome-weighted integrative clustering algorithm that integrates patient outcome data during the clustering process to identify cancer subtypes with prognostic significance [17]. The algorithm constructs an outcome-associated distance matrix that downweights features with no relevance to the outcome, thereby enabling accurate identification of cancer subtypes with significant survival differences. Simultaneously, adopting K-fold cross-validation, the survClust method avoids “overfitting” that occurs when using survival data in both model construction and prognosis evaluation [17].

In this study, we aim to identify LGG phenotypic subtypes using the survClust method based on preoperative multimodal MRI data from the First Hospital of Shanxi Medical University (FHSXMU) and Shanxi Provincial People’s Hospital (SPPH), and validate them in The Cancer Genome Atlas (TCGA)/The Cancer Imaging Archive (TCIA) dataset. Furthermore, we will explore biomarkers associated with LGG phenotypic subtypes, revealing the main driving factors of disease development and facilitating targeted interventions and prognostic evaluation in LGG patients.

## Methods

**Fig. 1:**
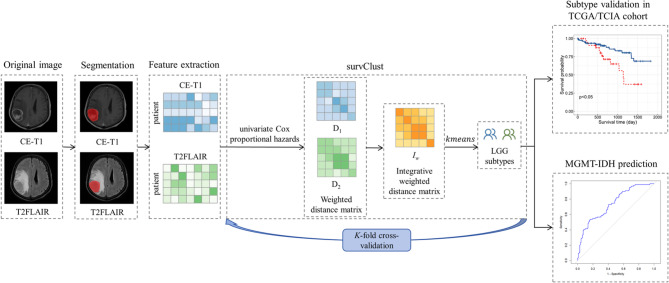
The overview of this study. We first extracted CE-T1 and T2FLAIR features from brain MRI images of LGG patients from FHSXMU/SPPH cohort and TCGA/TCIA cohort. In the FHSXMU/SPPH cohort, we identified two LGG phenotypic subtypes using SurvClust method based on multimodal MRI data. Then we performed predictive analysis of IDH mutations combined with MGMT promoter methylation, using significant MRI features identified between the subtypes. The identified phenotypic subtypes were subsequently validated in an external dataset (the TCGA/TCIA cohort) using SurvClust method based on significant MRI features identified between the subtypes

Statistical analyses were performed using R software (version 4.3.2). The significance level $\:\alpha\:=0.05$. The conceptual framework of this study is presented in Fig. 1.

### Patients

This study collected glioma patients from hospitals, including the FHSXMU and SPPH (344 cases), and the TCGA [18] (https://portal.gdc.cancer.gov/)/TCIA [19] (https://nbia.cancerimagingarchive.net/nbia-search/) project (208 cases). The inclusion criteria for LGG patients of this study are as follows: (i) pathologically confirmed WHO grade Ⅱ and grade Ⅲ glioma according to the 2021 WHO standards; (ii) availability of preoperative three-week MRI data, including CE-T1 and T2FLAIR images; (iii) clear genotype of isocitrate dehydrogenase (IDH) and O6-methylguanine-DNA methyltransferase (MGMT) promoter methylation status; (iv) follow-up time exceeded 2 years or occurred an endpoint event. Overall survival is calculated from the time of postoperative pathological diagnosis until death or the last follow-up. The exclusion criteria were as follows: (1) targeted treatment (including conservative treatment, radiotherapy and chemotherapy) had been received before surgery; (2) severe poor image quality or image motion artifacts; (3) lack of postoperative follow-up or loss to follow-up. Ultimately, 280 patients met these criteria, including 162 patients from the FHSXMU/SPPH cohort and 118 patients from the TCGA/TCIA cohort.

### MRI feature extraction and quantification

In the FHSXMU/SPPH cohort, all preoperative MRI images were acquired from 3.0-T scanners. The detailed scanning parameters are shown in the supplementary information (1). In the TCGA/TCIA cohort, preoperative MRI was acquired from TCIA project. Since the images from the TCGA/TCIA database come from different MRI devices and acquisition protocols, and there are also differences in MRI devices and acquisition protocols between the FHSXMU and SPPH datasets, we resampled all MRI images to ensure the spatial resolution and intensity of the images are comparable. This process eliminates the interference caused by inconsistent spatial resolution due to different MRI device models, and we performed intensity normalization using the min-max normalization method. This process was implemented using the SimpleITK library (https://github.com/SimpleITK/SimpleITK) in Python (version 3.10.4).

The T2FLAIR images were registered to the corresponding CE-T1 images through affine transformation using the FSL software (FMRIB Software Library, FSL, https://fsl.fmrib.ox.ac.uk/fsl/fslwiki/FSL). We chose CE-T1 as the primary reference for segmentation due to its high spatial resolution and clinical utility in defining tumor boundaries, which is critical for surgical planning and volumetric assessment. For the enhanced tumor ROI, the border was defined by the enhanced rim, and the tumor region of interest (ROI) was outlined on CE-T1 images and then transferred to T2FLAIR images. For unenhanced tumors, the tumor intensity on CE-T1 MRI images was lower than the surrounding peritumoral edema, and the ROI was similarly outlined on CE-T1 images and transferred to T2FLAIR. If the tumor border was unclear on both CE-T1 and T2FLAIR MRI images, DWI and ADC MRI images (if available) were used to help define the boundaries. Two radiologists, with varying years of experience in brain MRI diagnosis and without prior knowledge of the patients’ clinical data or other imaging results, imported the CE-T1 images into ITK-SNAP software (http://www.itksnap.org, version 3.8.0) for image segmentation. They first converted the images from DICOM to NIFTI format and then performed image segmentation. The tumor ROI was outlined slice by slice either manually or with semi-automated computer-assisted methods, to obtain the three-dimensional volume of interest (VOI). Subsequently, the resulting VOI images were imported into FAE software [20] (https://github.com/salan668/FAE, version 0.5.6) for MRI feature extraction. The extracted features include 18 first order features, 14 shape features, and 75 texture features. Texture features consist of 24 Gray Level Co-occurrence Matrix (GLCM) features, 14 Gray Level Dependence Matrix (GLDM) features, 16 Gray Level Run-Length Matrix (GLRLM) features, 16 Gray Level Size Zone Matrix (GLSZM) features, and 5 Neighboring Gray Tone Difference Matrix (NGTDM) features. Simultaneously, six types of image filters—wavelet transform, square, square root, logarithm, exponential, and gradient—were used for preprocessing the original images.

Finally, in the FHSXMU/SPPH cohort, a total of 1688 MRI features were extracted from each of the CE-T1 and T2FLAIR images. Two variables (T1c_lbp-3D-m1_firstorder_Minimum and T1c_lbp-3D-m2_firstorder_Minimum) of CE-T1 were excluded due to their values were entirely zero, retaining 1686 features. Similarly, two variables (FLAIR_lbp-3D-m1_firstorder_Minimum and FLAIR_lbp-3D-m2_firstorder_Minimum) of T2FLAIR were excluded because 98.15% of their values were zero, leaving 1686 features. We obtained the same MRI features from the TCGA/TCIA cohort. Subsequently, both CE-T1 and T2FLAIR data were standardized for further analysis.

To evaluate feature reproducibility, MRI features extracted from ROIs were tested for intra- and inter-observer (intraclass correlation coefficient, ICC) agreement using repeated segmentations by one radiologist (> 3-month interval) on 30 randomly selected LGG MRI cases. The MRI features demonstrated excellent reproducibility, with intra-observer ICC values (median = 0.88, IQR: [0.86–0.91]) and inter-observer ICCs (median = 0.85, IQR: [0.82–0.87]). Additionally, 86.7% of tested features showed ICC > 0.75.

###  IDH genotype and MGMT promoter methylation status data

For the FHSXMU/SPPH cohort, IDH mutation status was analyzed by Sanger sequencing, while MGMT promoter methylation was evaluated via pyrosequencing [21]. The details of test methods are described in our prior publication [22]. For patients in the TCGA/TCIA cohort, IDH genotype and MGMT promoter methylation status data were obtained from the Genomic Data Commons Data Portal.

### Acquisition and processing of mRNA expression data

The mRNA expression data of the TCGA/TCIA cohort were downloaded from the TCGA project. We excluded non-protein-coding genes, averaged duplicate genes and samples (retaining only one for each duplicate), and removed genes and samples with over 30% zero expression. To refine the distribution of gene expression data for more robust statistical analysis, we transformed gene expression values by $\:{\text{log}}_{2}(x+1)$. Finally, we obtained the mRNA expression data including 19,938 genes.

### The survClust method

The survClust algorithm (https://github.com/arorarshi/survClust) constructs a weighted distance matrix that down-weights MRI features unrelated to the outcome of interest [17]. Let $\:{X}_{m}$ be the $\:m\text{t}\text{h}$ data type with dimensions $\:{N}_{m}\times\:{p}_{m}$, where $\:{N}_{m}$ is the number of samples and $\:{p}_{m}$ is the number of features, with rows representing samples and columns representing features. The univariable Cox proportional hazards model fitted for each feature is calculated as:

$$\:\text{h}\left(t|{x}_{p}\right)={h}_{0}\times\:exp\left({x}_{j}^{T}*\beta\:\right)$$

Here, $\:t$ denotes survival time, $\:{x}_{j}$ refers to the $\:j$-th column of matrix $\:X$ with length $\:N$, $\:{h}_{0}$ is the baseline hazard function, $\:\beta\:$ represents the regression coefficient, and $\:exp\left(\beta\:\right)$ indicates the hazard ratio (HR).

The absolute value of the HR on the logarithmic scale from a univariate Cox proportional hazards model, fitted for the survival outcome (time) with each feature, is considered as the feature weight $\:{w}_{k}$. Then we can obtain the feature-weighted matrix $\:W=\text{d}\text{i}\text{a}\text{g}\left\{{w}_{1},{w}_{2},\cdots\:,{w}_{k}\right\}$. The weighted distance matrix $\:{d}_{w}$, for a pair of two samples $\:\text{a}$ and $\:\text{b}$, is calculated as follows:

$$\:{d}_{w}\left(a,b\right)=\sqrt{{\left(a-b\right)}^{T}W\left(a-b\right)}$$

Then we calculate the integrated weighted distance matrix:

$${I}_{w}=\sum_{m=1}^{M}{\gamma}_{m}{D}_{m}$$

$\:{D}_{m}(m=\text{1,2},\cdots\:M)$ is the $\:m\text{t}\text{h}$ weighted distance matrix, and $\:{\gamma\:}_{m}=\frac{1}{M}\forall\:m$. $\:{I}_{w}$ is then clustered by k-means [23]. To avoid overfitting and achieve more generalizable solutions, survClust employs K-fold cross-validation [17]. Final subtype labels were determined by consensus voting across 50 rounds of cross-validation.

In this study, we obtained the integrated weighted distance based on multimodal MRI data through the aforementioned steps. The cluster initialization was performed using the k-means + + algorithm [24], with 100 random starts to ensure stability. The clustering process was set to run for a maximum of 100 iterations, with convergence defined as centroid displacements < 1.00e-5 between consecutive iterations. We used 3-fold cross-validation, selected based on survClust’s validation framework [17], which demonstrates strong subtype stability with smaller sample sizes.

### Estimating the optimal number of clusters

Clustering Prediction Index (CPI) estimates the optimal cluster number ($\:{k}_{1}$) by resampling based cross-validation technique [25]. It works by splitting the data into training and test sets, repeating this to generate multiple pairs. A clustering algorithm trains on the training data to find cluster centers, which then assign test samples to the nearest cluster. The CPI is computed as the average within-cluster sum of squares across all test samples, calculated as follows:

$$\text{CPI}\left(k\right)=\frac1m\sum_{i=1}^m\sum_{x\in X_{n_2\times p_i}^i}\left\|x-C_i^{\left(k1\right)}\left(x\right)\right\|^2$$

Here, $\:m$ denotes the number of data splits, and $\:{C}_{i}^{\left(k1\right)}\left(x\right)$ represents the assigned cluster centroid for the test sample $x$.

Gap-statistics determines the optimal number of clusters ($\:{k}_{2}$) by comparing the observed within-cluster sum of squares($\:{W}_{k2}$) with its expected value under null reference distribution [26]. The Gap-statistics is maximized as follows:

$$\begin{aligned}\:{\text{Gap}}_{n}\left(k\right)=&{E}_{n}^{*}\left\{\text{log}\left(\sum\:_{r=1}^{k2}\frac{1}{2{n}_{r}}\sum\:_{i,i{\prime\:}\in\:{C}_{r}}{d}_{ii{\prime\:}}\right)\right\}\\&-\text{log}\left(\sum\:_{r=1}^{k2}\frac{1}{2{n}_{r}}\sum\:_{i,i{\prime\:}\in\:{C}_{r}}{d}_{ii{\prime\:}}\right)\end{aligned}$$

Here, $\:n$ represents the sample size, $\:{C}_{r}$ indicates each cluster, $\:i$ and $\:i{\prime\:}$ are the observations within the cluster, and $d$ measures their squared Euclidean distance.

In this study, the optimal cluster number was selected at the maximum sum of CPI and Gap-statistics [27].

### Identification and evaluation of LGG phenotypic subtypes

For the FHSXMU/SPPH cohort, we identified LGG phenotypic subtypes based on multimodal MRI data using the survClust method. The Kruskal-Wallis *H*-test was used to select MRI features with significant differences between the different subtypes in the FHSXMU/SPPH cohort, followed by a false discovery rate (FDR) adjustment ($\:{P}_{adj}<0.05$). Subsequently, these selected features were matched by name with MRI features from the TCGA/TCIA cohort. Based on these matched MRI features, the survClust method was used to subtype patients in the TCGA/TCIA cohort.

With a five-year cutoff, we analyzed survival differences among patients of different subtypes using Kaplan-Meier survival analysis and log-rank test. We conducted differential analysis for various factors between different subtypes, with continuous variables analyzed using the Kruskal-Wallis *H*-test and categorical variables evaluated using the Chi-Square test. In the FHSXMU/SPPH cohort, we employed a multivariate Cox proportional hazards model to evaluate prognostic differences among LGG phenotypic subtypes and identify the high-risk subtype, adjusting for age, gender, and pathological stage. To ensure the reliability of the Cox model, we performed an evaluation of all variables in the Cox model using the Schoenfeld residuals test, and calculated the Events Per Variable (EPV).

### Differential and enrichment analysis of MRI features

For the selected differential MRI features after the Kruskal-Wallis *H*-test with an FDR-adjusted *P*-value ($\:{P}_{adj}<0.05$), we conducted enrichment analysis for CE-T1 and T2FLAIR features across LGG phenotypic subtypes using the hypergeometric test with an FDR-adjusted *P*-value ($\:{P}_{adj}<0.05$).

To select features significantly enriched for distinct LGG phenotypic subtypes, a feature was considered over-expressed or under-expressed if its standardized Z-scores exceeded 0.6 or lower than − 0.6. For each subtype, the hypergeometric test was applied to evaluate significant enrichment of either over-expressed or under-expressed features. To identify most representative features of a given subtype, stringent criteria were applied: only features significantly enriched in at least 2/3 of samples within a subtype and in fewer than 1/3 of samples in any other subtype were selected.

### Immune infiltration analysis and pathway activity analysis

We conducted immune infiltration analysis and pathway activity analysis using mRNA expression data from the TCGA/TCIA cohort. Using the ‘BIOR’ package, we estimated tumor cell composition and performed Kruskal-Wallis *H*-tests with an FDR-adjusted *P*-value ($\:{P}_{adj}<0.05$) to identify immune cells exhibiting significantly different infiltration levels among different subtypes.

The pathway activity analysis was conducted through the following steps: (1) differentially expressed genes (DEGs) between different subtypes in the TCGA/TCIA cohort were identified using the “limma” package (version 3.58.1). (2) Using the “decoupleR” package (version 2.9.7), the gene weights and *P* values for 14 pathways were retrieved from the PROGENy database and the top 100 most significant genes (ranked by *P* value) were retained for per pathway. (3) A multivariate linear model (MLM) was applied to the DEGs to estimate pathway activity score. For each sample in the TCGA/TCIA cohort, the MLM predicted gene expression based on the pathway-gene interaction weights across 14 pathways. The resulting slope t-values represented the pathway activity scores, where positive t-values indicated pathway activation and negative t-values suggested inactivation.

### Predictive modeling of IDH mutation combined with MGMT promoter methylation

IDH mutation and MGMT promoter methylation are frequently observed in LGG [28]. Previous studies have demonstrated that the glioma patients with IDH mutation combined with MGMT promoter methylation have a longer overall survival (OS) and/or progression-free survival (PFS) [29, 30]. Therefore, precise identification of patients with combined IDH mutation and MGMT promoter methylation is critical for treatment decision-making and prognosis evaluation in glioma patients. The predictive model based on multimodal MRI for IDH mutation and MGMT promoter methylation status in glioma has potential as a non-invasive auxiliary diagnostic tool [9, 31].

In this study, patients with both MGMT promoter methylation and IDH mutation were grouped together as “MGMTmet&IDHmut” group, while all others were assigned to “Others” group, labeled as the MGMT-IDH variable.

Both CE-T1 and T2FLAIR data contain numerous features, most of which may be unrelated to predicting MGMT-IDH. Therefore, feature selection is necessary to optimize predictive model performance. We combined the selected differential MRI features after the Kruskal-Wallis *H*-test into a matrix, and conducted a stability feature selection on the matrix using a penalized logistic regression [32]. The matrix was randomly split into training and testing datasets with an 8:2 ratio. Then, we used L1-penalized logistic regression on the training data to select features, while selected the optimal lambda value using 10-fold cross-validation. We recorded the feature selection results from 100 random splits (seed = 1-100) of the samples, and select MRI features with a frequency of occurrence ≥ 10% as the final selected features. Ridge regression (L2 penalty) can serve as an alternative shrinkage method, but it retains all features, potentially keeping noise variables. In our study, we prefer L1-penalized logistic regression for feature selection, as it can shrink the coefficients of irrelevant features to zero, achieving sparsity and interpretability. This aligns directly with our goal of predicting MGMT-IDH status using a minimal set of MRI features.

Then, using final selected MRI features as predictors, we constructed MGMT-IDH prediction models based on partial least squares with the genetic algorithm (GA-fKPLS) [33], logistic regression (LR) [34], random forest (RF) [35], support vector machine (SVM) [36], *k*-nearest neighbor (*k*NN) [37].

In this study, patients from the FHSXMU/SPPH cohort were split into two non-overlapping subsets, with 80% used for training and 20% for testing. To enhance the stability and reproducibility of the results, the random splitting process was repeated 1000 times under different seed numbers (seed numbers = 1-1000). To validate the model’s effectiveness, we conducted comprehensive external validation using the TCGA/TCIA cohort, employing the same feature selection and modeling methods as for the FHSXMU/SPPH cohort.

We employed eight evaluation criteria to assess the predictive performance of the five models, including the area under the curve (AUC), sensitivity (Se), specificity (Sp), accuracy (ACC), Youden index, F-measure, Matthews correlation coefficient (MCC), and G-means. The final performance of each model was evaluated based on the average values of these eight metrics across the 1000 independent data splits. Among these, the MCC and AUC were the main criteria used for model evaluation, as they provide a more comprehensive evaluation of model performance. To further illustrate the performance of the five models, we used one-way analysis of variance followed by Dunnett’s multiple comparison test to compare the highest AUC value with the AUC values of the other four models.

## Results

### Patient characteristics

Basic characteristics of patients from the FHSXMU/SPPH cohort and the TCGA/TCIA cohort are shown in Table 1.

**Table 1:** Patient characteristics in the FHSXMU/SPPH cohort and TCGA/TCIA cohort

Characteristic	FHSXMU/SPPH cohort (*n* = 162)	TCGA/TCIA cohort (*n* = 118)
Sex *n* (%)		
Female	86(53.09)	55(46.61)
Male	76(46.91)	63(53.39)
Age *n* (%)		
<35 years	76(46.91)	27(22.88)
≥35 years	86(53.09)	91(77.12)
Pathological grade *n* (%)		
WHO II	82(50.62)	54(45.76)
WHO III	80(49.38)	64(54.24)
MGMT promoter status *n* (%)		
Unmethylation	44(27.16)	18(15.25)
Methylation	118(72.84)	100(84.75)
IDH genotype *n* (%)		
Wild type	73(45.06)	24(20.34)
Mutation	89(54.94)	94(79.66)
Vital status *n* (%)		
Death	106(65.43)	32(27.12)
Survival	56(34.57)	86(72.88)
Tumor volume (*cm*^*3*^, *mean*±*SD*)	38.10±35.13	42.92±53.59

### Identification of two LGG phenotypic subtypes

**Fig. 2:**
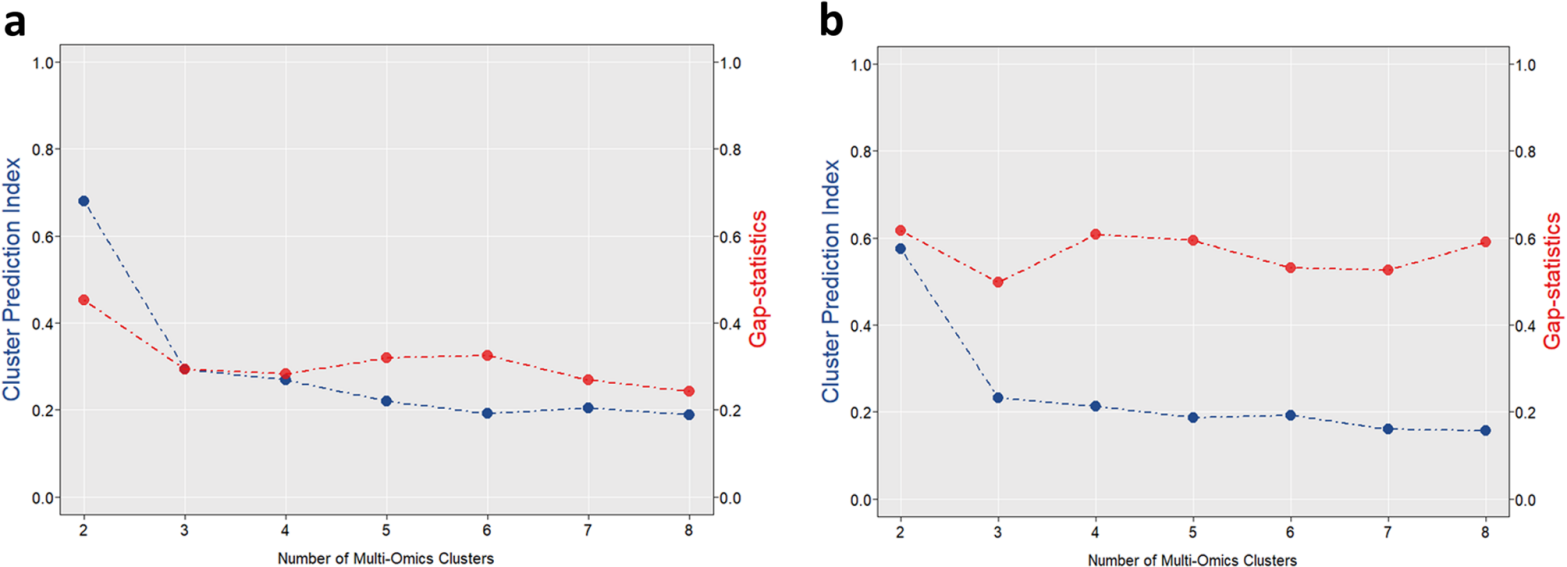
Results of CPI and Gap-statistic from 2 to 8 clusters in the FHSXMU/SPPH cohort (**a**) and TCGA/TCIA cohort (**b**)

Since the sum of CPI and Gap-statistic was maximum when the number of clusters was 2, we decided 2 as the optimal number of clusters for the FHSXMU/SPPH cohort (Fig. 2a). In the FHSXMU/SPPH cohort, based on multimodal MRI data, two LGG phenotypic subtypes (subtype 1 with 51 patients and subtype 2 with 111 patients) were successfully identified using the survClust method. Subtype 1 had 9 deaths out of 51 patients (17.6%), while Subtype 2 had 47 deaths out of 111 patients (42.3%).

1333 CE-T1 features and 1273 T2FLAIR features with significant differences between the two subtypes were selected in the FHSXMU/SPPH cohort. The MRI features of TCGA/TCIA cohort were subsequently matched to the 2606 selected features based on their names. Because the sum of CPI and Gap-statistic was maximum when the number of clusters was 2, we selected 2 as the optimal number of clusters for the TCGA/TCIA cohort (Fig. 2b). Based on these matched MRI features, two subtypes were identified by survClust method, with 84 patients assigned to subtype 1 and 34 patients to subtype 2. In the TCGA/TCIA cohort, there were 20 deaths among the 84 patients in Subtype 1 and 12 deaths among the 34 patients in Subtype 2.

**Fig. 3:**
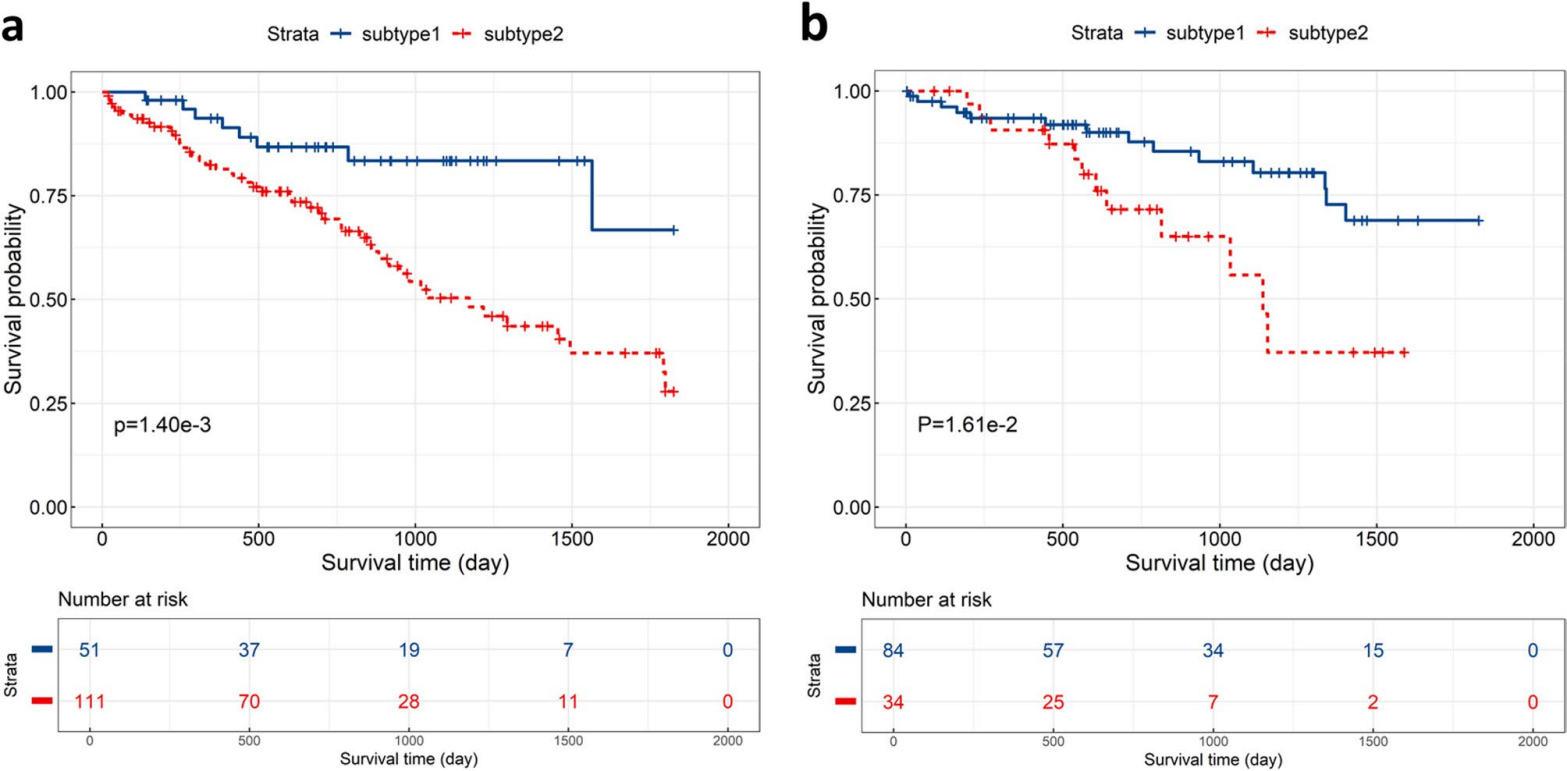
Kaplan-Meier curves of the LGG phenotypic subtypes in the FHSXMU/SPPH cohort (**a**) and TCGA/TCIA cohort (**b**)

For both FHSXMU/SPPH cohort and TCGA/TCIA cohort, Kaplan-Meier survival analysis and log-rank test demonstrated significant prognostic difference ($\:P<0.05$) between the two subtypes (Fig. 3).

### Evaluation of LGG phenotypic subtypes

As presented in Table 2, in the FHSXMU/SPPH cohort, pathological grade, MGMT promoter status, IDH genotype, vital status, and tumor volume were statistically significant between the two subtypes ($\:P<0.05$). The results of the multivariate Cox regression analysis, adjusted for age, gender, and pathological stage in the FHSXMU/SPPH cohort, are shown in Table 3. Patients in subtype 2 were 2.553 times higher risk of death compared to patients in subtype 1 ($\:P<0.05$). Patients with WHO Ⅲ grade were 1.775 times higher risk of death compared to patients with WHO Ⅱ grade ($\:P<0.05$). The Schoenfeld residuals test for all variables in the Cox model confirmed no significant time-dependent trends (Table S2).

**Table 2:** Baseline clinical and molecular data of FHSXMU/SPPH cohort

Characteristic	Subtype 1 (*n* = 51)	Subtype 2 (*n* = 111)	df	χ^2^/H	*P*
Sex *n* (%)					
Female	31(60.78)	55(49.55)			
Male	20(39.22)	56(50.45)	1	1.35	0.25
Age *n* (%)					
<35 years	8(15.69)	22(19.82)			
≥ 35 years	43(84.31)	89(80.18)	1	0.17	0.68
Pathological grade *n* (%)					
WHO II	35(68.63)	47(42.34)			
WHO III	16(31.37)	64(57.66)	1	8.64	**3.30e-3**
MGMT promoter status *n* (%)					
Unmethylation	8(15.69)	36(32.43)			
Methylation	43(84.31)	75(67.57)	1	4.14	**0.04**
IDH genotype *n* (%)					
Wild type	15(29.41)	58(52.25)			
Mutation	36(70.59)	53(47.75)	1	6.47	**0.01**
Vital status *n* (%)					
Death	9(17.65)	47(42.34)			
Survival	42(82.35)	64(57.66)	1	8.36	**3.83e-3**
Tumor volume (*cm*^*3*^, *mean*±*SD*)	10.30±8.11	50.87±35.41	1	83.34	**< 2.2e-16**
MGMT-IDH *n* (%)					
MGMTmet&IDHmut	35(68.63)	44(39.64)			
Others	16(31.37)	67(60.36)	1	10.62	**1.12e-3**

A *P*<0.05 was considered statistically significant. “df” represents degrees of freedom:$$Chi-Square\:test\:degrees\:of\:freedom\:=$$$$\:(number\:of\:subtypes\:-\:1)\:\times\:\:(number\:of\:categories\:in\:variable\:2\:-\:1);$$$$Kruskal-Wallis\:H-test\:degrees\:of\:freedom\:=\:number\:of\:subtypes\:-\:1$$

**Table 3:** The multivariable Cox analysis for FHSXMU/SPPH cohort

Variable	B	Wald	SE	*P*	HR(95% CI)
Subtype					
Subtype 2	0.937	6.276	0.374	**0.012**	2.553(1.226–5.315)
Sex					
Female	0.159	0.33	0.277	0.566	1.173(0.681–2.020)
Age					
≥ 35 years	0.556	1.83	0.411	0.176	1.744(0.779–3.902)
Pathological grade					
WHO III	0.574	3.86	0.292	**0.049**	1.775(1.001–3.145)

Using subtype 1, male, < 35 years old and WHO II grade as the reference group. A$\:P<0.05$was considered statistically significant. Cox model$\:EPV\:=\:14$

As shown in Table 4, in the TCGA/TCIA cohort, IDH genotype and tumor volume were significantly different between the two subtypes ($\:P<0.05$).

**Table 4:** Baseline clinical and molecular data of TCGA/TCIA cohort

Characteristic	Subtype 1 (*n* = 84)	Subtype 2 (*n* = 34)	df	χ^2^/H	*P*
Sex *n* (%)					
Female	39(46.43)	16(47.06)			
Male	45(53.57)	18(52.94)	1	0.00	1.00
Age *n* (%)					
<35 years	23(27.38)	4(11.76)			
≥ 35 years	61(72.62)	30(88.24)	1	2.52	0.11
Pathological grade *n* (%)					
WHO II	37(44.05)	17(50.00)			
WHO III	47(55.95)	17(50.00)	1	0.15	0.70
MGMT promoter *n* (%)					
Unmethylation	9(10.71)	9(26.47)			
Methylation	75(89.29)	25(73.53)	1	3.51	0.06
IDH genotype *n* (%)					
Wild type	11(13.10)	13(38.24)			
Mutation	73(86.90)	21(61.76)	1	7.95	**4.80e-3**
Vital status *n* (%)					
Death	20(23.81)	12(35.29)			
Survival	64(76.19)	22(64.71)	1	1.09	0.29
Tumor volume (*cm*^*3*^, *mean*±*SD*)	57.46±57.40	7.00±5.12	1	47.88	**4.53e-12**

A$$\:P<0.05$$was considered statistically significant. “df” represents degrees of freedom:$$\:Chi-Square\:test\:degrees\:of\:freedom\:=\:(number\:of\:subtypes\:-\:1)\:\times\:\:(number\:of\:categories\:in\:variable\:2\:-\:1);$$$$Kruskal-Wallis\:H-test\:degrees\:of\:freedom\:=\:number\:of\:subtypes\:-\:1$$

### Result of the differential and enrichment analysis of MRI features

**Fig. 4:**
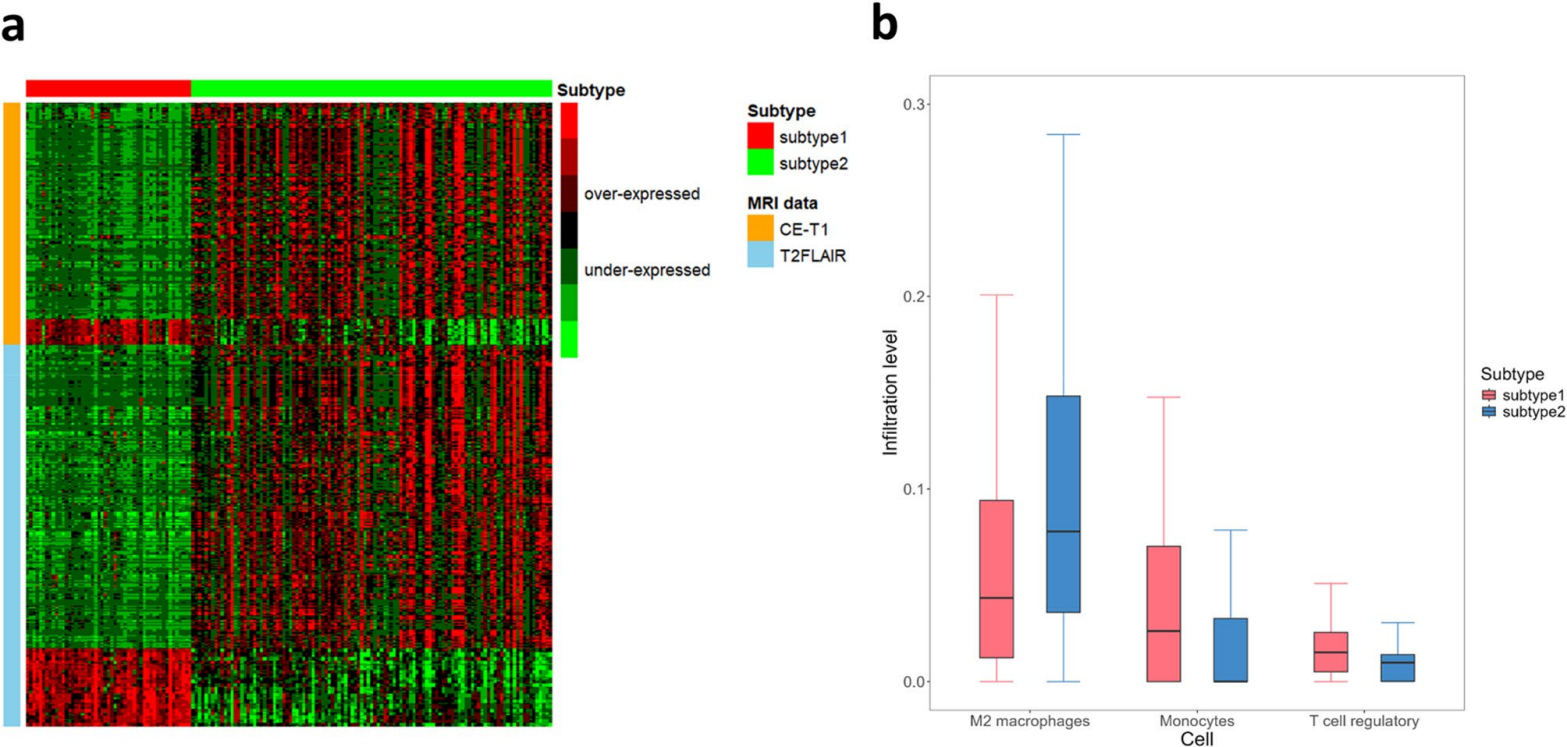
Downstream analysis results. (**a**) Heatmap of identified differentially enriched MRI features across the 2 subtypes of FHSXMU/SPPH cohort. The color scale represents the standardized Z-scores of MRI features (green: under-expressed, $\:Z\le\:-0.6$; red: over-expressed, $\:Z\ge\:0.6$). (**b**) Boxplot of three significantly different immune cell infiltration levels across different subtypes of TCGA/TCIA cohort

In the FHSXMU/SPPH cohort, 469 differentially expressed MRI features were identified across different subtypes (Fig. 4a), including 182 features of CE-T1 and 287 features of T2FLAIR. Among the 182 CE-T1 features, 19 features were over-expressed, while 163 features were under-expressed. For the 287 T2FLAIR features, 59 features were over-expressed, and 228 features were under-expressed. These features provide crucial imaging-based evidence for the precise identification of molecular subtypes of glioma. For example, the under-expressed texture feature of MRI features – Gray Level Non-Uniformity measures the variability of intensity values of gray level in an image, with a lower value indicating more homogeneity in intensity values [38]. The 10mm_GLRLM_Gray Level Non-Uniformity in glioblastoma patients may reflect internal hypoxic and necrotic areas, along with the heterogeneous distribution of tumor cell subtypes, which collectively impact tumor behavior, including growth, invasiveness, and treatment response [39].

### Pathway activity analysis in the TCGA/TCIA cohort

As presented in Table 5, three pathways were identified with statistically significant differences in activity between two subtypes ($\:{P}_{adj}<0.05$) in the TCGA/TCIA cohort. Specifically, JAK-STAT, TNF-α, and p53 were significantly less active in subtype 1 compared to subtype 2.

**Table 5:** The result of pathway activity analysis in the TCGA/TCIA cohort

Pathway	*t*	*P*_adj_
Androgen	0.43	0.66
EGFR	−1.82	0.19
Estrogen	1.34	0.31
Hypoxia	1.40	0.31
JAK-STAT	−14.93	**5.56e-49**
MAPK	−1.10	0.36
NF-κB	1.29	0.31
PI3K	1.07	0.36
TGF-β	0.99	0.38
TNF-α	−2.99	**0.02**
TRAIL	−2.02	0.15
VEGF	1.28	0.31
Wnt	−0.50	0.66
p53	−2.91	**0.02**

Using subtype 2 as the reference subtype. A $\:{P}_{adj}<0.05$ was considered statistically significant

### Immune infiltration analysis in the in the TCGA/TCIA cohort

In the TCGA/TCIA cohort, we have analyzed the infiltration levels of 11 immune cells. As shown in Table 6, the infiltration levels of M2 macrophages, Monocytes and T cell regulatory (Tregs) were significantly different between two subtypes ($\:{P}_{adj}<0.05$). As presented in Fig. 4b, the infiltration level of M2 macrophages was higher in patients of subtype 2 than in those of subtype 1, whereas Monocytes and Tregs showed significantly lower infiltration levels in subtype 2.

**Table 6:** Result of immune infiltration level analysis in the TCGA/TCIA cohort

Cell type	*P*_adj_
M2 macrophages	**0.02**
Monocytes	**0.02**
T cell regulatory	**0.04**
uncharacterized cell	0.25
Myeloid dendritic cell	0.52
Neutrophil	0.67
M1 macrophages	0.68
NK cell	0.68
T cell CD4+ (non-regulatory)	0.68
T cell CD8+	0.68
B cell	0.93

A $\:{P}_{adj}<0.05$ was considered statistically significant

### Performance comparison of predictive models for IDH mutation combined with MGMT promoter methylation in the FHSXMU/SPPH cohort

“MGMTmet&IDHmut” group includes 79 patients, while “Others” group includes 83 patients. The MGMT-IDH variable was significantly different between the two subtypes in the FHSXMU/SPPH cohort (Table 2). We identified 23 features from CE-T1 and 19 features from T2FLAIR by stability feature selection process.

**Fig. 5:**
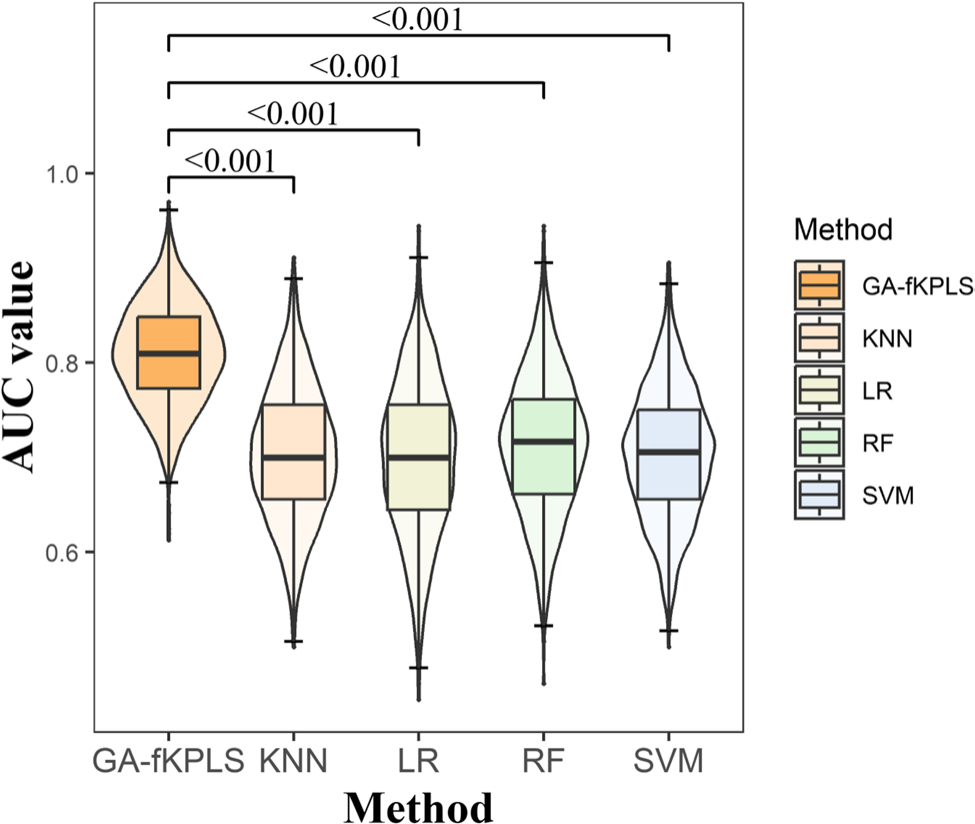
Boxplot of AUC values distribution for 5 predictive models in the FHSXMU/SPPH cohort. Y-axis represents the AUC value

As shown in Table 7, the GA-fKPLS model outperformed the other models, as it achieved the best performance in AUC, ACC, Youden index, MCC, and G-means. The distribution of AUC values for the five models is shown in Fig. 5, and the AUC value of the GA-fKPLS model was significantly higher than other models ($\:P<0.05$). In the TCGA/TCIA cohort, the GA-fKPLS model also outperformed the other four predictive models, with detailed results presented in Supplementary Information (3).

**Table 7:** Model performance summary of 5 predictive models in the FHSXMU/SPPH cohort

Models	AUC	Se	Sp	ACC	Youden	F-measure	MCC	G-means
GA-fKPLS	**0.809**	0.786	0.654	**0.727**	**0.440**	0.759	**0.450**	**0.713**
LR	0.699	0.744	0.655	0.703	0.399	0.730	0.406	0.692
RF	0.713	0.799	0.627	0.721	0.427	0.756	0.442	0.703
SVM	0.703	**0.843**	0.564	0.716	0.407	**0.763**	0.433	0.683
KNN	0.704	0.747	**0.660**	0.707	0.407	0.734	0.415	0.697

To demonstrate the clinical significance of GA-fKPLS model in predicting patients with IDH mutation combined with MGMT promoter methylation, we selected the prediction results from a single sample random split, with an $\:MCC=0.440$ (close to the $\:{MCC}_{mean}=0.450$). There were 32 patients in the test set of this random split.

**Fig. 6:**
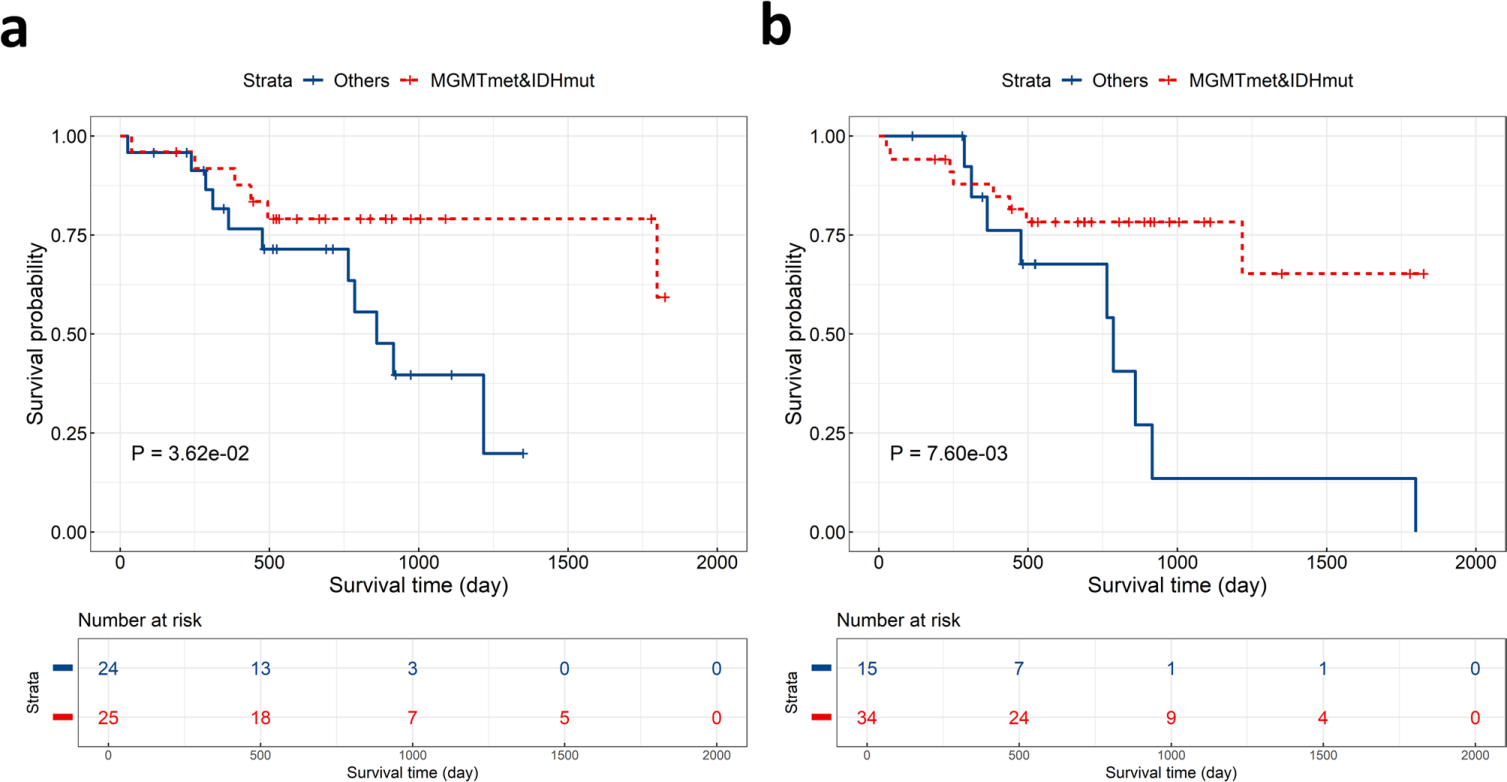
Kaplan-Meier survival curves of patients in MGMTmet&IDHmut group and Others group in the test set. (**a**) The survival curve for the two MGMT-IDH groups is based on the original data. (**b**) The survival curve for the two MGMT-IDH groups is based on the GA-fKPLS predicted results

In the test set, based on the original data and GA-fKPLS model predicted results respectively, we used Kaplan-Meier survival analysis and log-rank test to analyze survival differences between the two MGMT-IDH groups. As shown in Fig. 6, the survival prognoses of the two MGMT-IDH groups were significantly different ($\:P<0.05$), and the survival curve based on the GA-fKPLS predicted results was close to it derived from the original data. The above results indicate that the GA-fKPLS model exhibits good performance in predicting IDH mutation combined with MGMT promoter methylation in LGG patients.

## Discussion

Low-grade glioma is a clinically heterogeneous group of primary brain tumors with variable treatment responses to current treatment regimens. Consequently, accurate identification of phenotypic subtypes prior to treatment may play a critical role in guiding therapeutic decisions and monitoring prognosis. Using the outcome-weighted integrative clustering method – survClust, we successfully identified two LGG phenotypic subtypes based on preoperative multimodal MRI data (CE-T1 and T2FLAIR) from the FHSXMU/SPPH cohort, which were further validated in the TCGA/TCIA cohort. These two LGG phenotypic subtypes showed distinct prognostic outcomes, clinical characteristics, and molecular features. These findings may contribute to a better understanding of LGG heterogeneity and could potentially inform future efforts in clinical decision-making and precision-targeted therapy development.

In the FHSXMU/SPPH cohort, the mortality risk of subtype 2 was 2.553 times higher than that of subtype 1, and there were significant differences between the subtypes in terms of prognosis, pathological grade, tumor volume, MGMT promoter methylation, IDH mutation, and imaging feature expression levels. Patients with glioma of subtype 1, which had a better survival outcome, exhibited lower pathological grades, smaller tumor volumes, and a higher proportion of cases with both MGMT promoter methylation and IDH mutation, consistent with previous research [11, 28, 40]. While our analysis identified 469 differentially expressed MRI features across subtypes in the FHSXMU/SPPH cohort, it is important to note that the observed associations between these MRI features and pathological characteristics remain inferential rather than mechanistic. Although prior literature suggests plausible biological correlations for certain features [38, 39], the computational nature of MRI features means they represent mathematical abstractions of texture patterns rather than direct measurements of specific biological processes. This represents a fundamental limitation in interpreting radiomic signatures, as the precise biological underpinnings of most features have yet to be fully elucidated through histopathological validation studies. The GA-fKPLS model, constructed based on preoperative multimodal MRI data of LGG, achieved significantly higher AUC values compared to the other four machine learning models, indicating its superior predictive performance for IDH mutation combined with MGMT promoter methylation. The GA-fKPLS model achieves a high sensitivity of 0.917, which is crucial for reliably identifying patients with IDH mutation combined with MGMT promoter methylation. In settings where minimizing false negatives is a priority, such as screening or early intervention, the GA-fKPLS model shows potential as an auxiliary tool for clinical assessment.

In the TCGA/TCIA cohort, two subtypes exhibit significant differences in survival probability, which are associated with signaling pathway activities and levels of immune infiltration. We identified 4 differential pathways (JAK-STAT, TNF-α, p53) and 3 distinct immune cell infiltrates (M2 macrophages, Tregs, Monocytes) in LGG patients. The JAK-STAT signaling pathway directly modulates communication between transmembrane receptors and the nucleus, affecting various physiological functions such as cell proliferation, differentiation, apoptosis, immune regulation, and hematopoiesis [41]. Now, increasing evidence suggests that TNF-α pathway is one of the major mediators of cancer-associated inflammation and acts as a tumor-promoting factor, involved in all stages of tumor development, including transformation, proliferation, angiogenesis, invasion, and metastasis [42]. The TNF-α pathway activation is often associated with poor prognosis, and clinical trials of TNF-α antagonists have been encouraging and have shown promising results [43, 44]. The transcription factor p53 is an important regulator of the cell cycle and is considered one of the most crucial tumor suppressors [45]. It has been widely accepted that the loss of p53 function plays an important role in glioma tumorigenesis [46, 47]. Previous studies have demonstrated that M2 macrophages promotes immunosuppression and proliferation of LGG [48]. Regulatory T cells play a significant role in the suppression of antitumor immunity in human glioma. In this study, we found that high infiltration levels of regulatory T cells were associated with favorable prognosis in LGG [49]. Some studies have demonstrated that regulatory T cells suppress the body’s anti-tumor immune response, and their infiltration is associated with poor prognosis [50, 51]. However, the tumor microenvironment of brain gliomas is complex, exhibiting significant differences across various grades and subtypes of gliomas [52]. In glioblastoma, tumor cells can secrete chemokines such as C-C motif chemokine ligand 22 (CCL22), which binds to the C-C chemokine receptor type 4 (CCR4) receptor on the surface of Tregs, thereby recruiting Tregs and enhancing immunosuppression [53]. In contrast, in LGG, the immunosuppressive effect of Tregs may be relatively weak, allowing the body’s immune surveillance to potentially inhibit tumor progression. Additionally, the patient’s molecular characteristics and treatment modalities can influence the infiltration and function of Tregs [49, 54, 55]. For example, in IDH-mutant gliomas, Treg infiltration is reduced, and the degree of immunosuppression is lower, which is often correlated with a better prognosis. Furthermore, since this study relies on mRNA expression data from TCGA/TCIA and lacks supporting gene expression data from the FHSXMU/SPPH cohort, the reliability of the Treg-related findings requires further validation. Future research should integrate single-cell RNA sequencing and immunohistochemical methods to comprehensively explore the functional differences of Tregs across LGG subtypes. Although previously the brain was once thought to be immune-privileged, recent studies have revealed that it may be accessible to immune system, suggesting that immunotherapies may offer a viable treatment option for central nervous system (CNS) tumors, including glioblastoma [56]. However, initial trials of immunotherapies in glioma have demonstrated only limited efficacy [57, 58]. The exploration of immunotherapy for LGG remains a great challenge. Notably, as shown in Table 4, subtype 2 had a smaller median tumor volume than subtype 1, despite being associated with a poorer prognosis in the TCGA/TCIA cohort. This counterintuitive observation may be attributed to the aggressive molecular characteristics of subtype 2, such as IDH-wildtype status and unmethylated MGMT. These features are associated with diffuse infiltration and rapid disease progression, even when tumors appear smaller on imaging [59]. Such molecular traits may allow these gliomas to remain undetected until advanced stages or to progress aggressively without a proportional increase in volume. Furthermore, tumor volume alone may not reflect the underlying molecular or immunological heterogeneity that drives prognosis in LGG. For instance, components of the immune microenvironment, such as M2 macrophages, have been shown to independently contribute to poor outcomes, irrespective of tumor size [60]. Consequently, relying solely on tumor volume to assess aggressiveness may be inadequate. Instead, imaging phenotypes should be evaluated alongside molecular and immunological data to achieve a more comprehensive understanding of prognosis. Our findings suggest that multimodal MRI data may help identify prognosis-associated pathway activities and immune infiltration patterns, providing preliminary insights that could inform future research into targeted therapies and immunotherapies for LGG.

Undoubtedly, several limitations need to be recognized in this work. First, the clinical applicability of our MRI-based subtypes and GA-fKPLS model still requires validation through prospective studies. Current guidelines (WHO 2021, NCCN guidelines) still consider histopathology and molecular testing as the core basis for treatment decisions [61]. These models should only be considered as supplementary imaging information for reference. Second, Due to the characteristics of the GA-fKPLS methodology, the model outputs continuous discrimination values rather than probabilities, which means that standard calibration curves, Brier scores, and Decision Curve Analysis (DCA) cannot be directly computed. This limitation may hinder the model’s clinical translatability. Third, we focused solely on two MRI modalities (CE-T1 and T2FLAIR), the most commonly used imaging modality for LGG, to allow the broad applicability and use of the model in the clinical setting. Integrating advanced MRI modalities, such as diffusion-weighted imaging (dMRI) and perfusion-weighted imaging (PWI), is an important direction for our future research. These modalities have great potential to enrich the phenotypic characterization of gliomas by providing complementary information on tumor cellularity, metabolic activity, and vascular heterogeneity. Larger studies will become possible as newer imaging techniques, such as magnetic resonance spectroscopy (MRS) and positron emission tomography (PET) imaging become more widely available in the future [62, 63]. Quantitative features derived from advanced imaging modalities and techniques may further enhance the explanation of our subtypes or reveal new associations with biological molecules. Fourth, in the FHSXMU/SPPH cohort, pathway activity and immune infiltration analyses were unable to be performed owing to unavailable gene expression data, which may limit the comprehensive exploration of the biological drivers underlying the identified phenotypic subtypes.

## Conclusions

In summary, two LGG phenotypic subtypes with different survival outcomes and molecular features were identified using the outcome-weighted integrative clustering method based on preoperative multimodal MRI data. This approach may aid future risk stratification for LGG patients, pending prospective validation, thereby offering supplementary information for clinical decision-making and prognosis evaluation.

## Supplementary Information

Below is the link to the electronic supplementary material.

Supplementary Material 1

## Data Availability

The clinical information and image data of FHSXMU/SPPH cohort are not publicly available due to patient privacy and ethical restrictions but are available from the corresponding author on reasonable request. The clinical information and image data of TCGA/TCIA cohort used are all publicly available: TCGA (https:/portal.gdc.cancer.gov) and TCIA (https:/nbia.cancerimagingarchive.net/nbia-search/). In support of research reproducibility, the radiomic feature matrices for the FHSXMU/SPPH cohort, including features derived from each modality (CE-T1 and T2FLAIR), are deposited in a public repository (https:/github.com/yq20010209/MRI-Radiomics-Features-of-Low-Grade-Glioma).

## Abbreviations

LGG: Low-grade Glioma
MRI: Magnetic Resonance Imaging
FHSXMU: First Hospital of Shanxi Medical University
SPPH: Shanxi Provincial People’s Hospital
TCGA: The Cancer Genome Atlas
TCIA: The Cancer Imaging Archive
CE-T1: Post-Contrast Enhanced T1-Weighted
T2FLAIR: T2-Weighted Fluid Attenuation Inversion Recovery
IDH: Isocitrate Dehydrogenase
MGMT: O6-Methylguanine-DNA Methyltransferase
FDR: False Discovery Rate
GA-fKPLS: Fused Kernel Partial Least Squares with the Genetic Algorithm
LR: Logistic Regression
RF: Random Forest
SVM: Support Vector Machine
*k*NN: *k*-Nearest Neighbor
AUC: Area Under the Curve
WHO: World Health Organization
TR: Repetition Time
TE: Echo Time
FOV: Field of View
FSL: FMRIB Software Library
ROI: Region of Interest
VOI: Volume of Interest
GLCM: Gray Level Co-occurrence Matrix
GLDM: Gray Level Dependence Matrix
GLRLM: Gray Level Run Length Matrix
GLSZM: Gray Level Size Zone Matrix
NGTDM: Neighboring Gray Tone Difference Matrix
EPV: Events Per Variable
ICC: Intraclass Correlation Coefficient
HR: Hazard Ratio
CPI: Clustering Prediction Index
DEGs: Differentially Expressed Genes
MLM: Multivariate Linear Model
OS: Overall Survival
PFS: Progression-Free Survival
Se: Sensitivity
Sp: Specificity
MCC: Matthews Correlation Coefficient
Tregs: T Cell Regulatory
DCA: Decision Curve Analysis
CCL22: C-C Motif Chemokine Ligand 22
CCR4: C-C Chemokine Receptor Type 4
CNS: Central Nervous System
dMRI: Diffusion-Weighted Imaging
PWI: Perfusion-Weighted Imaging
MRS: Magnetic Resonance Spectroscopy
PET: Positron Emission Tomography

## References

[1] Forst DA, Nahed BV, Loeffler JS, Batchelor TT. Low-grade gliomas. Oncologist. 2014;19:403–13. 24664484 10.1634/theoncologist.2013-0345 PMC3983820

[2] Chen B, Liang T, Yang P, Wang H, Liu Y, Yang F, et al. Classifying lower grade glioma cases according to whole genome gene expression. Oncotarget. 2016;7:74031–42. 27677590 10.18632/oncotarget.12188 PMC5342033

[3] Network CGAR. Comprehensive, integrative genomic analysis of diffuse lower-grade gliomas. New Engl J Med. 2015;372:2481–98. 26061751 10.1056/NEJMoa1402121 PMC4530011

[4] Gittleman H, Sloan AE, Barnholtz-Sloan JS. An independently validated survival nomogram for lower-grade glioma. Neurooncol. 2020;22:665–74. 10.1093/neuonc/noz191 PMC7229246 31621885

[5] Van den Bent MJ. Interobserver variation of the histopathological diagnosis in clinical trials on glioma: a clinician’s perspective. Acta Neuropathol. 2010;120:297–304. 20644945 10.1007/s00401-010-0725-7 PMC2910894

[6] Shukla G, Alexander GS, Bakas S, Nikam R, Talekar K, Palmer JD, et al. Advanced magnetic resonance imaging in glioblastoma: a review. Chin Clin Oncol. 2017;6:40. 28841802 10.21037/cco.2017.06.28

[7] Sanai N, Berger MS. Operative techniques for gliomas and the value of extent of resection. Neurotherapeutics. 2009;6:478–86. 19560738 10.1016/j.nurt.2009.04.005 PMC5084184

[8] Fukuda A, Queiroz LDS, Reis F. Gliosarcomas: magnetic resonance imaging findings. Arquivos de Neuro-Psiquiatria. 2020;78:112–20. 32022137 10.1590/0004-282X20190158

[9] Kim M, Jung SY, Park JE, Jo Y, Park SY, Nam SJ, et al. Diffusion- and perfusion-weighted MRI radiomics model may predict isocitrate dehydrogenase (IDH) mutation and tumor aggressiveness in diffuse lower grade glioma. Eur Radiol. 2020;30:2142–51. 31828414 10.1007/s00330-019-06548-3

[10] Tan Y, Zhang S, Wei J, Dong D, Wang X, Yang G, et al. A radiomics nomogram may improve the prediction of IDH genotype for Astrocytoma before surgery. Eur Radiol. 2019;29:3325–37. 30972543 10.1007/s00330-019-06056-4

[11] Haldar D, Kazerooni AF, Arif S, Familiar A, Madhogarhia R, Khalili N, et al. Unsupervised machine learning using K-means identifies radiomic subgroups of pediatric low-grade gliomas that correlate with key molecular markers. Neoplasia. 2023;36:100869. 36566592 10.1016/j.neo.2022.100869 PMC9803939

[12] Gevaert O, Mitchell LA, Achrol AS, Xu J, Echegaray S, Steinberg GK, et al. Glioblastoma multiforme: exploratory radiogenomic analysis by using quantitative image features. Radiology. 2014;273:168–74. 24827998 10.1148/radiol.14131731 PMC4263772

[13] Itakura H, Achrol AS, Mitchell LA, Loya JJ, Liu T, Westbroek EM, et al. Magnetic resonance image features identify glioblastoma phenotypic subtypes with distinct molecular pathway activities. Sci Transl Med. 2015;7:r138–303. 10.1126/scitranslmed.aaa7582 PMC4666025 26333934

[14] Sathyanarayanan A, Gupta R, Thompson EW, Nyholt DR, Bauer DC, Nagaraj SH. A comparative study of multi-omics integration tools for cancer driver gene identification and tumour subtyping. Brief Bioinform. 2020;21:1920–36. 31774481 10.1093/bib/bbz121 PMC7711266

[15] Coretto P, Serra A, Tagliaferri R. Robust clustering of noisy high-dimensional gene expression data for patients subtyping. Bioinformatics. 2018;34:4064–72. 29939219 10.1093/bioinformatics/bty502

[16] Rappoport N, Shamir R. Multi-omic and multi-view clustering algorithms: review and cancer benchmark. Nucleic Acids Res. 2018;46:10546–62. 30295871 10.1093/nar/gky889 PMC6237755

[17] Arora A, Olshen AB, Seshan VE, Shen R. Pan-cancer identification of clinically relevant genomic subtypes using outcome-weighted integrative clustering. Genome Med. 2020;12:1–13. 10.1186/s13073-020-00804-8 PMC7716509 33272320

[18] Tomczak K, Czerwinska P, Wiznerowicz M. The cancer genome atlas (TCGA): an immeasurable source of knowledge. Contemp Oncol (Pozn). 2015;19:A68–77. 25691825 10.5114/wo.2014.47136 PMC4322527

[19] Zanfardino M, Pane K, Mirabelli P, Salvatore M, Franzese M. TCGA-TCIA impact on radiogenomics cancer research: A systematic review. Int J Mol Sci. 2019;20:6033. 31795520 10.3390/ijms20236033 PMC6929079

[20] Song Y, Zhang J, Zhang Y, Hou Y, Yan X, Wang Y, et al. Feature explorer (FAE): a tool for developing and comparing radiomics models. PLoS One. 2020;15:e237587. 10.1371/journal.pone.0237587 PMC7431107 32804986

[21] Dunn J, Baborie A, Alam F, Joyce K, Moxham M, Sibson R, et al. Extent of MGMT promoter methylation correlates with outcome in glioblastomas given temozolomide and radiotherapy. Br J Cancer. 2009;101:124–31. 19536096 10.1038/sj.bjc.6605127 PMC2713697

[22] Yang G, Sha Y, Wang X, Tan Y, Zhang H. Radiomics profiling identifies the incremental value of MRI features beyond key molecular biomarkers for the risk stratification of High-Grade gliomas. Contrast Media Mol I. 2022;2022:8952357. 10.1155/2022/8952357 PMC8967578 35386727

[23] Hartigan JA, Wong MA, Algorithm AS. A k-means clustering algorithm. J Royal Stat Soc Ser C (Applied Statistics). 1979;136:28:100–8.

[24] Yang J, Wang Y, Yao X, Lin C. Adaptive initialization method for k-means algorithm. Front Artif Intell. 2021;4:740817. 34901837 10.3389/frai.2021.740817 PMC8656690

[25] Chalise P, Fridley BL. Integrative clustering of multi-level ‘omic data based on non-negative matrix factorization algorithm. PLoS One. 2017;12:e176278. 10.1371/journal.pone.0176278 PMC5411077 28459819

[26] Tibshirani R, Walther G, Hastie T. Estimating the number of clusters in a data set via the gap statistic. J R Stat Soc Series B Stat Methodol. 2001;63:411–23.

[27] Lu X, Meng J, Zhou Y, Jiang L, Yan F. MOVICS: an R package for multi-omics integration and visualization in cancer subtyping. Bioinformatics. 2020;36:5539–41. 10.1093/bioinformatics/btaa1018 33315104

[28] Leu S, von Felten S, Frank S, Vassella E, Vajtai I, Taylor E, et al. IDH/MGMT-driven molecular classification of low-grade glioma is a strong predictor for long-term survival. Neurooncol. 2013;15:469–79. 10.1093/neuonc/nos317 PMC3607260 23408861

[29] Sha Y, Yan Q, Tan Y, Wang X, Zhang H, Yang G. Prediction of the molecular subtype of IDH mutation combined with MGMT promoter methylation in gliomas via radiomics based on preoperative MRI. Cancers. 2023;15:1440. 36900232 10.3390/cancers15051440 PMC10001198

[30] Zhang G, Tai P, Fang J, Chen A, Chen X, Cao K. Molecular subtypes based on centrosome-related genes can predict prognosis and therapeutic responsiveness in patients with low-grade gliomas. Front Oncol. 2023;13:1157115. 37051542 10.3389/fonc.2023.1157115 PMC10083401

[31] Chen Y, Liao Y, Li P, Jin W, Fang J, Huang J, et al. A systematic review and meta-analysis of deep learning and radiomics in predicting MGMT promoter methylation status in glioblastoma: efficacy, reliability, and clinical implications. Displays. 2025:103072.

[32] Meinshausen N, Bühlmann P. Stability selection. J R Stat Soc Series B Stat Methodol. 2010;72:417–73.

[33] Yang H, Cao H, He T, Wang T, Cui Y. Multilevel heterogeneous omics data integration with kernel fusion. Brief Bioinform. 2020;21:156–70. 30496340 10.1093/bib/bby115

[34] Stoltzfus JC. Logistic regression: a brief primer. Acad Emerg Med. 2011;18:1099–104. 21996075 10.1111/j.1553-2712.2011.01185.x

[35] Breiman L. Random forests. Mach Learn. 2001;45:5–32.

[36] Hearst MA, Dumais ST, Osuna E, Platt J, Scholkopf B. Support vector machines. Ieee Intell Syst their Appl. 1998;13:18–28.

[37] Steinbach M, Tan P. kNN: k-nearest neighbors. The top ten algorithms in data mining. Chapman and Hall/CRC. 2009. pp. 165–76.

[38] Chen Y, Qi Y, Li T, Lin A, Ni Y, Pu R, et al. A more objective PD diagnostic model: integrating texture feature markers of cerebellar gray matter and white matter through machine learning. Front Aging Neurosci. 2024;16:1393841. 38912523 10.3389/fnagi.2024.1393841 PMC11190310

[39] Chen Y, Lin H, Sun J, Pu R, Zhou Y, Sun B. Texture feature differentiation of glioblastoma and solitary brain metastases based on tumor and tumor-brain interface. Acad Radiol. 2025;32:400–10. 39217081 10.1016/j.acra.2024.08.025

[40] Tanaka K, Sasayama T, Mizukawa K, Takata K, Sulaiman NS, Nishihara M, et al. Combined IDH1 mutation and MGMT methylation status on long-term survival of patients with cerebral low-grade glioma. Clin Neurol Neurosurg. 2015;138:37–44. 26276726 10.1016/j.clineuro.2015.07.019

[41] Xin P, Xu X, Deng C, Liu S, Wang Y, Zhou X, et al. The role of JAK/STAT signaling pathway and its inhibitors in diseases. Int Immunopharmacol. 2020;80:106210. 31972425 10.1016/j.intimp.2020.106210

[42] Balkwill F. Tumour necrosis factor and cancer. Nat Rev Cancer. 2009;9:361–71. 19343034 10.1038/nrc2628

[43] Garber K. First results for agents targeting cancer-related inflammation. Jnci: Journal of the National Cancer Institute. 2009;101:1110–2. 19671776 10.1093/jnci/djp266

[44] Wu Y, Zhou BP. TNF-a/NF-kB/Snail pathway in cancer cell migration and invasion. Brit J Cancer. 2010;102:639–44. 20087353 10.1038/sj.bjc.6605530 PMC2837572

[45] Lane D, Levine A. p53 research: the past thirty years and the next thirty years. Cold Spring Harb Perspect Biol. 2010;2:a893. 10.1101/cshperspect.a000893 PMC2982174 20463001

[46] Muñoz DM, Tung T, Agnihotri S, Singh S, Guha A, Zadeh G, et al. Loss of p53 cooperates with K-ras activation to induce glioma formation in a region-independent manner. Glia. 2013;61:1862–72. 24038521 10.1002/glia.22563

[47] Muñoz DM, Guha A. Mouse models to interrogate the implications of the differentiation status in the ontogeny of gliomas. Oncotarget. 2011;2:590–8. 21896959 10.18632/oncotarget.319 PMC3248213

[48] Zhu Y, Song Z, Wang Z, Chen G. Protective prognostic biomarkers negatively correlated with macrophage M2 infiltration in Low-Grade glioma. J Oncol. 2022;2022:3623591. 35432538 10.1155/2022/3623591 PMC9012619

[49] Richardson LG, Nieman LT, Stemmer-Rachamimov AO, Zheng XS, Stafford K, Nagashima H, et al. IDH-mutant gliomas harbor fewer regulatory T cells in humans and mice. Oncoimmunology. 2020;9:1806662. 32923170 10.1080/2162402X.2020.1806662 PMC7458656

[50] Chongsathidkiet P, Jackson C, Koyama S, Loebel F, Cui X, Farber SH, et al. Sequestration of T cells in bone marrow in the setting of glioblastoma and other intracranial tumors. Nat Med. 2018;24:1459–68. 30104766 10.1038/s41591-018-0135-2 PMC6129206

[51] Humphries W, Wei J, Sampson JH, Heimberger AB. The role of Tregs in glioma-mediated immunosuppression: potential target for intervention. Neurosurg Clin N Am. 2010;21:125. 19944972 10.1016/j.nec.2009.08.012 PMC2786818

[52] Quail DF, Joyce JA. The microenvironmental landscape of brain tumors. Cancer Cell. 2017;31:326–41. 28292436 10.1016/j.ccell.2017.02.009 PMC5424263

[53] Chang AL, Wainwright DA, Dey M, Han Y, Lesniak MS. CCR4 + regulatory T cells progressively accumulate in the presence of leukocyte-derived CCL22/CCL17 in an experimental model of glioblastoma multiforme. Cancer Res. 2014;74:1091.24351288

[54] Lin H, Liu C, Hu A, Zhang D, Yang H, Mao Y. Understanding the immunosuppressive microenvironment of glioma: mechanistic insights and clinical perspectives. J Hematol Oncol. 2024;17:31. 38720342 10.1186/s13045-024-01544-7 PMC11077829

[55] Pachocki CJ, Hol EM. Current perspectives on diffuse midline glioma and a different role for the immune microenvironment compared to glioblastoma. J Neuroinflammation. 2022;19:276. 36403059 10.1186/s12974-022-02630-8 PMC9675250

[56] Louveau A, Smirnov I, Keyes TJ, Eccles JD, Rouhani SJ, Peske JD, et al. Structural and functional features of central nervous system lymphatic vessels. Nature. 2015;523:337–41. 26030524 10.1038/nature14432 PMC4506234

[57] Young JS, Dayani F, Morshed RA, Okada H, Aghi MK. Immunotherapy for high-grade gliomas: a clinical update and practical considerations for neurosurgeons. World Neurosurg. 2019;124:397–409. 30677574 10.1016/j.wneu.2018.12.222 PMC6642850

[58] Reardon DA, Brandes AA, Omuro A, Mulholland P, Lim M, Wick A, et al. Effect of nivolumab vs bevacizumab in patients with recurrent glioblastoma: the checkmate 143 phase 3 randomized clinical trial. JAMA Oncol. 2020;6:1003–10. 32437507 10.1001/jamaoncol.2020.1024 PMC7243167

[59] Ceccarelli M, Barthel FP, Malta TM, Sabedot TS, Salama SR, Murray BA, et al. Molecular profiling reveals biologically discrete subsets and pathways of progression in diffuse glioma. Cell. 2016;164:550–63. 26824661 10.1016/j.cell.2015.12.028 PMC4754110

[60] Ren J, Xu B, Ren J, Liu Z, Cai L, Zhang X, et al. The importance of M1-and M2-polarized macrophages in glioma and as potential treatment targets. Brain Sci. 2023;13:1269. 37759870 10.3390/brainsci13091269 PMC10526262

[61] Nabors B, Portnow J, Hattangadi-Gluth J, Horbinski C. NCCN CNS tumor guidelines update for 2023. Neuro Oncol. 2023;25:2114–6. 37706665 10.1093/neuonc/noad169 PMC10708932

[62] Gallamini A, Zwarthoed C, Borra A. Positron emission tomography (PET) in oncology. Cancers. 2014;6:1821–89. 25268160 10.3390/cancers6041821 PMC4276948

[63] Wilson M, Andronesi O, Barker PB, Bartha R, Bizzi A, Bolan PJ, et al. Methodological consensus on clinical proton MRS of the brain: review and recommendations. Magn Reson Med. 2019;82:527–50. 30919510 10.1002/mrm.27742 PMC7179569

