# Supplementary material for: Phenotypic stratification of Low-grade Glioma using multimodal MRI via outcome-weighted integrative clustering
Source: BMC Neurol. 2025 Nov 4;25:452. doi: 10.1186/s12883-025-04420-0 (PMC12584545; doi:10.1186/s12883-025-04420-0)
Supplement: Supplementary file 1 — Supplementary Material 1 [file 12883_2025_4420_MOESM1_ESM.docx]

**Supplementary Materials for “Phenotypic stratification of Low-Grade Glioma using multimodal MRI via outcome-weighted integrative clustering”**

Qi Yang^1,4#^, Gaiqin Liu^1,4#^, Tong Wang^1,2^, Zhaoyang Xu^1,2^, Junyu Yan^1,2,5^, Ruiling Fang^1,4^, Yanhong Luo^1,4^, Hongmei Yu^1,4^, Yan Tan^3,5^, Hui Zhang^3,5^, Guoqiang Yang^3,5*^, Hongyan Cao^1,4*^

1 Department of Health Statistics, Shanxi Provincial Key Laboratory of Major Diseases Risk Assessment, School of Public Health, Shanxi Medical University, Taiyuan 030001, China

2 Academy of Medical Sciences, Shanxi Medical University, Taiyuan 030001, China

3 College of Medical Imaging, Shanxi Medical University, Taiyuan 030001, China

4 MOE Key Laboratory of Coal Environmental Pathogenicity and Prevention, Shanxi Medical University, Taiyuan 030001, China

5 Department of Radiology, First Hospital of Shanxi Medical University, Taiyuan 030001, China

^#^ These authors contributed equally to this work.

^*^ Correspondence:

Guoqiang Yang, Department of Radiology, First Hospital of Shanxi Medical University, Taiyuan 030001, China.

Email: [doctor_ygq@163.com](mailto:Doctor_ygq@163.com)

Hongyan Cao, Department of Health Statistics, Shanxi Provincial Key Laboratory of Major Diseases Risk Assessment, School of Public Health, Shanxi Medical University, Taiyuan 030001, China.

Email: [caohy@sxmu.edu.cn](mailto:caohy@sxmu.edu.cn)

**1. Supplementary table**

**Table S1** Radiomic features derived from the stability feature selection

| **CE-T1** | |
| --- | --- |
| 1 | T1c_original_glszm_SizeZoneNonUniformity |
| 2 | T1c_wavelet.LLH_glszm_LowGrayLevelZoneEmphasis |
| 3 | T1c_wavelet.LHH_glcm_Imc2 |
| 4 | T1c_wavelet.LLL_glszm_SmallAreaHighGrayLevelEmphasis |
| 5 | T1c_wavelet.LLH_glrlm_ShortRunHighGrayLevelEmphasis |
| 6 | T1c_squareroot_glcm_InverseVariance |
| 7 | T1c_log.sigma.1.mm.3D_glszm_SizeZoneNonUniformity |
| 8 | T1c_squareroot_glszm_SizeZoneNonUniformity |
| 9 | T1c_wavelet.HLH_glcm_Imc2 |
| 10 | T1c_wavelet.HLL_glszm_SmallAreaHighGrayLevelEmphasis |
| 11 | T1c_wavelet.HLL_glszm_ZoneEntropy |
| 12 | T1c_original_shape_Elongation |
| 13 | T1c_wavelet.LLH_glrlm_LowGrayLevelRunEmphasis |
| 14 | T1c_wavelet.LHL_firstorder_Variance |
| 15 | T1c_wavelet.LHL_glszm_ZoneEntropy |
| 16 | T1c_exponential_glszm_SmallAreaLowGrayLevelEmphasis |
| 17 | T1c_wavelet.LLH_gldm_LargeDependenceLowGrayLevelEmphasis |
| 18 | T1c_lbp.3D.m2_gldm_LargeDependenceLowGrayLevelEmphasis |
| 19 | T1c_wavelet.HLL_glcm_Imc2 |
| 20 | T1c_wavelet.HHH_glszm_ZoneEntropy |
| 21 | T1c_wavelet.HHL_glrlm_LongRunLowGrayLevelEmphasis |
| 22 | T1c_square_glszm_SmallAreaLowGrayLevelEmphasis |
| 23 | T1c_wavelet.LHL_firstorder_Skewness |
| **T2FLAIR** | |
| 1 | FLAIR_logarithm_glszm_SmallAreaLowGrayLevelEmphasis |
| 2 | FLAIR_logarithm_glszm_GrayLevelVariance |
| 3 | FLAIR_logarithm_glcm_InverseVariance |
| 4 | FLAIR_squareroot_glszm_SmallAreaLowGrayLevelEmphasis |
| 5 | FLAIR_wavelet.LHL_glrlm_ShortRunHighGrayLevelEmphasis |
| 6 | FLAIR_logarithm_glszm_LowGrayLevelZoneEmphasis |
| 7 | FLAIR_logarithm_firstorder_Minimum |
| 8 | FLAIR_wavelet.LHL_glcm_DifferenceVariance |
| 9 | FLAIR_wavelet.LHL_glszm_SizeZoneNonUniformity |
| 10 | FLAIR_wavelet.LHL_glrlm_LongRunLowGrayLevelEmphasis |
| 11 | FLAIR_original_glcm_ClusterProminence |
| 12 | FLAIR_wavelet.LLH_glcm_SumEntropy |
| 13 | FLAIR_wavelet.LLH_gldm_LargeDependenceLowGrayLevelEmphasis |
| 14 | FLAIR_wavelet.LLH_glcm_ClusterProminence |
| 15 | FLAIR_lbp.3D.m2_firstorder_Range |
| 16 | FLAIR_gradient_glcm_ClusterProminence |
| 17 | FLAIR_original_glcm_ClusterTendency |
| 18 | FLAIR_wavelet.HHL_firstorder_Mean |
| 19 | FLAIR_wavelet.LLH_glcm_MCC |

**Table S2** The Schoenfeld residuals test result of Cox model

| **Variable** | ***χ*^2^** | ***P*** |
| --- | --- | --- |
| Subtype | 0.061 | 0.805 |
| Sex | 1.605 | 0.205 |
| Age | 2.756 | 0.097 |
| Pathological grade | 0.288 | 0.592 |
| GLOBAL | 3.827 | 0.430 |

*Note.* A $P<0.05$ was considered statistically significant.

**2. Supplementary Information (1): Detailed scanning protocol for MRI in the First Hospital of Shanxi Medical University, and Shanxi Provincial People's Hospital**

Preoperative MRI was conducted using a 3.0-T scanner (Signa HDxt, GE Healthcare, USA) with an 8-channel array coil. The CE-T1 imaging was performed with a repetition time (TR) of 195 ms and an echo time (TE) of 4.76 ms. The T2FLAIR imaging was performed with a TR of 8,000 ms, TE of 95 ms, and an inversion time of 2,000 ms. The imaging slice thickness was 5.0 mm with 1.5 mm spacing. The field of view (FOV) was 240 × 240 mm², and the matrix size was 256 × 256. A gadolinium chelate contrast agent (0.1 mmol/kg) was applied for contrast-enhanced imaging.

**3. Supplementary Information (2) ：The hyper-parameters of GA-fKPLS**

We employed Gaussian kernel functions for both CE-T1 and T2-FLAIR data. The genetic algorithm parameters were set as follows: population size of 20 chromosomes, real-valued encoding, 80% crossover rate, 10% mutation rate, elitism preserving top 10% individuals, maximum of 3 generations, with convergence criteria being either reaching maximum generations or no improvement in the best fitness value (5-fold cross-validated Youden Index = sensitivity + specificity - 1) for 2 consecutive generations. Kernel weights ranged $(0,1)$ summing to 1, and Gaussian kernel parameters ranged $(0,\sqrt{p})$ (p represents the number of features in each modality).

**4. Supplementary Information (3) ：Performance comparison of predictive models for IDH mutation combined with MGMT promoter methylation in the FHSXMU/SPPH cohort**

The "MGMTmet&IDHmut" group consists of 88 patients, while the "Others" group includes 30 patients. Through the stability feature selection process, we identified 23 features from CE-T1 and 21 features from T2FLAIR. As shown in Table S3, the GA-fKPLS model outperformed the other models, achieving the highest performance in AUC, specificity, Youden index, and G-means. The AUC distribution for the five models is presented in Fig. S1, where the GA-fKPLS model's AUC was significantly higher than that of the other models (P<0.05).

**Table S3** Performance summary of 5 predictive models in the TCGA/TCIA cohort

| **Models** | **AUC** | **Se** | **Sp** | **ACC** | **Youden** | **F-measure** | **MCC** | **G-means** |
| --- | --- | --- | --- | --- | --- | --- | --- | --- |
| GA-fKPLS | **0.871** | 0.917 | **0.593** | 0.833 | **0.510** | 0.888 | 0.551 | **0.730** |
| LR | 0.677 | 0.785 | 0.567 | 0.731 | 0.352 | 0.811 | 0.341 | 0.651 |
| RF | 0.719 | 0.894 | 0.544 | 0.806 | 0.437 | 0.872 | 0.471 | 0.682 |
| SVM | 0.740 | 0.962 | 0.519 | **0.851** | 0.480 | **0.905** | **0.578** | 0.670 |
| KNN | 0.711 | **0.971** | 0.450 | 0.841 | 0.421 | 0.901 | 0.540 | 0.646 |


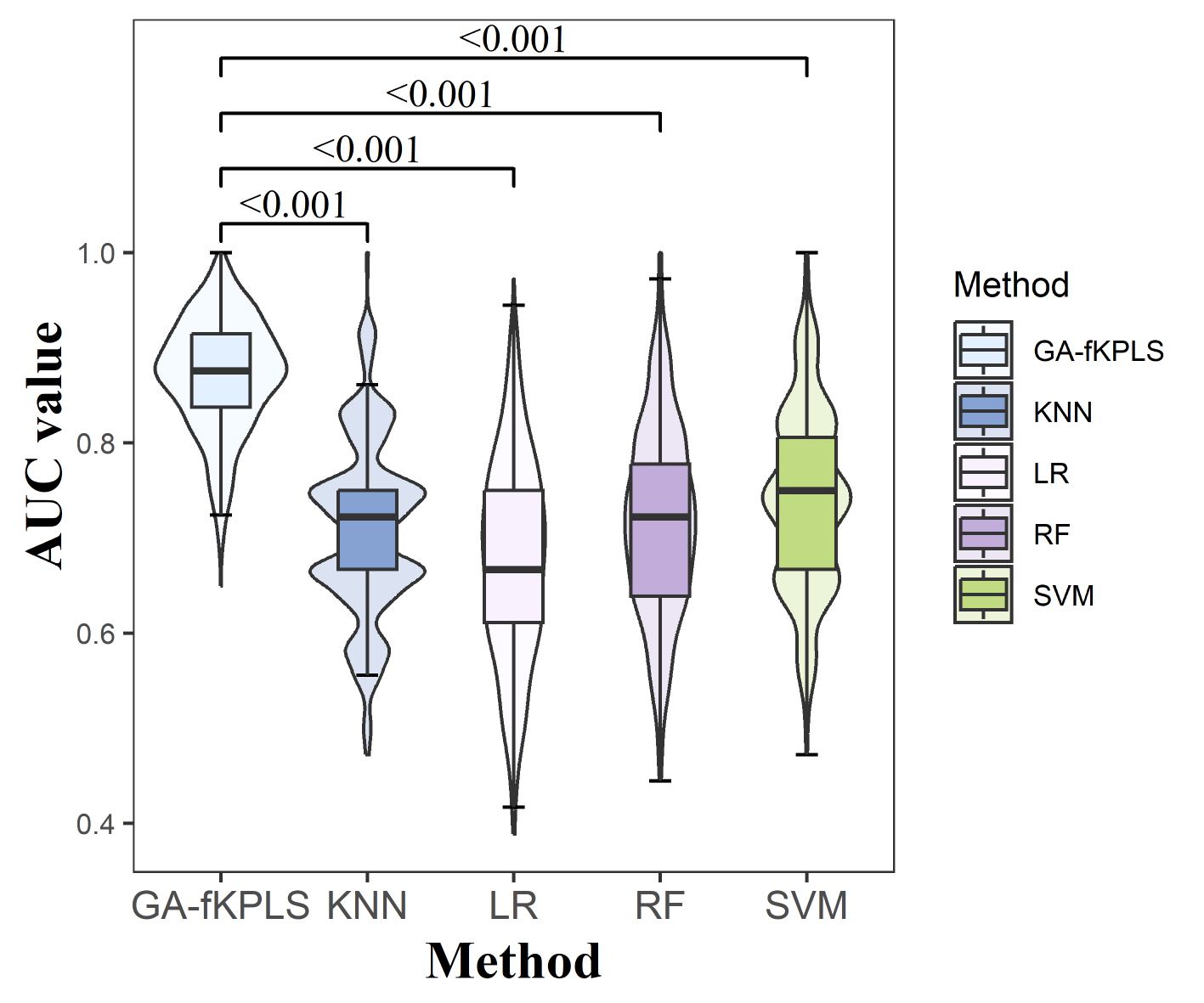


**Fig. S1** Boxplot of AUC values distribution for 5 predictive models in the TCGA/TCIA cohort. Y-axis represents the AUC value.
